# Impact of the rumen microbiome on milk fatty acid composition of Holstein cattle

**DOI:** 10.1186/s12711-019-0464-8

**Published:** 2019-05-29

**Authors:** Bart Buitenhuis, Jan Lassen, Samantha Joan Noel, Damian R. Plichta, Peter Sørensen, Gareth F. Difford, Nina A. Poulsen

**Affiliations:** 1 Department of Molecular Biology and Genetics, Center for Quantitative Genetics and Genomics, Aarhus University, Blichers Alle 20, P.O. Box 50, 8830 Tjele, Denmark; 2 Department of Animal Science, Aarhus University, Blichers Alle 20, P.O. Box 50, 8830 Tjele, Denmark; 3 Center for Biological Sequence Analysis, Denmark Technical University, 2800 Lyngby, Denmark; 4 Department of Food Science, Aarhus University, Blichers Alle 20, P.O. Box 50, 8830 Tjele, Denmark

## Abstract

**Background:**

Fatty acids (FA) in bovine milk derive through body mobilization, de novo synthesis or from the feed via the blood stream. To be able to digest feedstuff, the cow depends on its rumen microbiome. The relative abundance of the microbes has been shown to differ between cows. To date, there is little information on the impact of the microbiome on the formation of specific milk FA. Therefore, in this study, our aim was to investigate the impact of the rumen bacterial microbiome on milk FA composition. Furthermore, we evaluated the predictive value of the rumen microbiome and the host genetics on the composition of individual FA in milk.

**Results:**

Our results show that the proportion of variance explained by the rumen bacteria composition (termed microbiability or $h_{B}^{2}$) was generally smaller than that of the genetic component (heritability), and that rumen bacteria influenced most C15:0, C17:0, C18:2 n-6, C18:3 n-3 and CLA *cis*-9, *trans*-11 with estimated $h_{B}^{2}$ ranging from 0.26 to 0.42. For C6:0, C8:0, C10:0, C12:0, C16:0, C16:1 *cis*-9 and C18:1 *cis*-9, the variance explained by the rumen bacteria component was close to 0. In general, both the rumen microbiome and the host genetics had little value for predicting FA phenotype. Compared to genetic information only, adding rumen bacteria information resulted in a significant improvement of the predictive value for C15:0 from 0.22 to 0.38 (P = 9.50e−07) and C18:3 n-3 from 0 to 0.29 (P = 8.81e−18).

**Conclusions:**

The rumen microbiome has a pronounced influence on the content of odd chain FA and polyunsaturated C18 FA, and to a lesser extent, on the content of the short- and medium-chain FA in the milk of Holstein cattle. The accuracy of prediction of FA phenotypes in milk based on information from either the animal’s genotypes or rumen bacteria composition was very low.

**Electronic supplementary material:**

The online version of this article (10.1186/s12711-019-0464-8) contains supplementary material, which is available to authorized users.

## Background

The bovine rumen provides an anaerobic environment for the acquisition and promotion of a complex microbial community that consists of bacteria, archaea, fungi and protozoa, with bacteria being the most abundant [1]. The relative microbiome composition may differ between cows [2]. The rumen microbiome composition has been shown to have an important impact on the production of methane. Difford et al. [2] reported that variation in the relative rumen microbiome composition could explain up to 13% of the variation in total methane emission from dairy cattle and that the microbial community in the cows’ genetic make-up is associated with methane emission. The function of the metabolic activity of these microbial symbionts is to digest complex fibrous feed substrates into volatile fatty acids (VFA) and microbial proteins that are used directly by the host for maintenance, growth and lactation [3]. Furthermore, these fermentation products have an effect on milk composition [4, 5], thus the rumen microbial content has the potential to influence variation in host phenotypes [6].

Bovine milk contains many different fatty acids (FA), from essential FA such as linoleic (C18:2) and α-linolenic (18:3) FA to human health promoting FA such as conjugated linoleic acid (CLA) (C18:2) [7]. The short and medium chain FA (C4:0 to C14:0) are synthesized in the mammary gland. The long chain FA derive mainly from the feed but can be modified in the rumen and the mammary gland [8]. C16:0 can be derived both from feed and de novo synthesis in the mammary gland. To date, studies have focused on the genetic factors that affect the synthesis of FA in the milk and have shown that they are the main factors that influence the synthesis of saturated FA, whereas feed is more important for the synthesis of unsaturated FA [9–11]. Within the context of the digestion of feed by the microbial community in the rumen, it is interesting to investigate how much of the variation in milk FA can be explained by variation in the rumen microbiome.

Thus, our study focused on the influence of bacterial composition in the rumen microbiome on the synthesis of specific milk FA in Holstein cows by evaluating the value of the rumen microbiome in addition to the host genetics in predicting differences in the composition of individual FA phenotypes in milk.

## Methods

### Animals

Morning milk samples were collected from 339 Holstein cows from three different herds. Each herd had one to three visits within a short time period, during which rumen samples were collected from individual cows immediately after milking (one sample per cow). The cows were kept indoors, fed a total mixed ration (TMR) ad libitum, and milked individually using automated milking systems. They were divided into two groups according to parity: 120 cows for parity 1 and 209 cows for parity 2, and days in milk (DIM) ranged from 3 to 877. For all herds, a standardized TMR recipe was provided, which consisted primarily of rolled barley, corn silage, grass clover silage, rapeseed meal, soybean meal, and up to 3 kg of concentrate supplement given during milking. The procedures for collecting biological samples from the animals were approved by the Danish Veterinary and Food Administration under the Ministry of Environment and Food of Denmark and performed in accordance with the National Guidelines for Animal Experimentation and the guidelines of the Danish Animal Experimental Ethics Committee.

### Analysis of milk fatty acids

Analysis of milk FA was done as previously described in [12]. Fat percentage (FP) and protein percentage (PP) were determined on fresh whole-milk samples by infrared spectroscopy using a MilkoScan FT2 (Foss Analytical, Hillerød, Denmark). Cream was separated from skim milk by centrifugation (2643*g* for 30 min at 4 °C). Cream samples were stored at − 20 °C until analysis of FA composition by gas chromatography (GC), as described by Larsen et al. [13]. Peak areas for individual FA were calculated after GC separation. Fatty acids were identified and quantified based on external standards (Supelco 37 component FA methyl ester (FAME) mix, Supelco Inc., Bellefonte, PA and GLC 469 methyl ester standard, Nu-Chek Prep Inc., Elysian, MN), and values were expressed as weight proportion of total identified FA [12]. We measured the following FA: caproic acid (C6:0), caprylic acid (C8:0), capric acid (C10:0), lauric acid (C12:0), tridecylic acid (C13:0), myristic acid (C14:0), myristovaccenic acid (C14:1 *cis*-9), pentadecylic acid (C15:0), palmitic acid (C16:0), palmitoleic acid (C16:1 *cis*-9), margaric acid (C17:0), stearic acid (C18:0), oleic acid (C18:1*cis*-9), elaidic acid (C18:1 *trans*-11), linoleic acid (C18:2 n-6), α-linolenic acid (C18:3 n-3), and conjugated linoleic acid (CLA *cis*-9, *trans*-11). Since C18:1 *trans* isomers were not fully separated, the FA assigned as C18:1 *trans*-11 was a mixture of *trans*-10 and *trans*-11 isomers. Fatty acid measures are presented as proportions of total fat weight (wt%/wt%).

### Rumen sampling, DNA extraction and analysis of the bacterial community

Rumen sampling, DNA extraction, library generation and analysis of the bacterial community were performed as previously described in detail by Difford et al. [2]. Briefly, approximately 40 g of rumen content (both liquid and particle matter) were sampled using a flora rumen scoop and a representative subsample was immediately frozen at − 80 °C for DNA extraction [14]. The profile of the bacterial community was assessed by sequencing the V1–V3 region of the 16S rRNA gene. DNA extraction, sequence library construction and paired-end sequencing were performed by a commercial company (GATC Biotech, Constance, Germany). After quality control and processing of sequence reads, they were clustered into operational taxonomic units (OTU) based on a similarity threshold of 97% using the LotuS pipeline [15] via the UPARSE algorithm [16]. Low quality read ends were trimmed, and reads were quality filtered based on the following criteria: (1) a minimum average phred score of 27 and (2) a maximum accumulated error (E_max_) of 0.75, which represents the average number of errors per read, i.e. on average 0.75 wrongly sequenced basepairs per read. In order to focus on representative samples that had a sufficient sequencing depth to be able to describe less abundant taxa as well, we removed samples with less than 50,000 reads after quality control [17]. Finally, OTU that contained less than 10 sequences were filtered from the OTU table. Taxonomy was assigned to each OTU using the RDP classifier with a confidence level of 0.8 using greengenes (gg_13_8_otus) as the reference database (http://www.metagenomics.wiki/tools/16s/qiime/install/greengenes).

### Genotyping

Genomic DNA was extracted from ear tissue of the 339 Holstein cows and genotyped with the Bovine single nucleotide polymorphism (SNP) 50 beadchip (http://www.illumina.com/Documents/products/datasheets/datasheet_bovine_snp5O.pdf) using an Illumina^®^ Infinium II Multisample assay device. The iScan and the Beadstudio version 3.1 software were used to scan the SNP chips. SNPs were selected based on the following quality parameters: a minimum call rate of 80% for individuals and a minimum call rate of 95% for loci. SNPs with a minor allele frequency (MAF) lower than 1% were excluded. The quality of the SNPs was assessed using the Illumina GenCall data analysis software. Individuals with average GenCall scores lower than 0.65 were excluded as described in [18]. SNP positions were based on the *Bos taurus* genome assembly (*ARS*-*UCD1.2*) [19]. Finally, 39,121 SNPs remained for analysis.

### Cleaning of the animal data

We retained only the cows that had matched rumen samples, milk samples and genotypes (n = 310), and removed from these, those that were in lactation for more than 400 days, which resulted in 292 animals available for the analysis.

### Statistical analysis

#### Cluster analysis

In order to detect (biological) patterns in the bacterial OTU, a cluster analysis was performed based on the count data of the individual OTU. In total, 8515 bacterial OTU with 97% sequence similarity were identified. OTU with a total sequence count less than 10 were removed from the data, which resulted in 3055 bacterial OTU available for this analysis. The sequence count data of the bacterial genomes were centered (by subtracting the column means from their corresponding columns) and scaled (by dividing the (centered) columns by their standard deviations), resulting in the **B** matrix of 292 (number of cows) by 3055 (number of OUT). The distance matrix for cows based on **B** was calculated using the Dist function of the R package amap with the Pearson distance measure (https://cran.r-project.org/web/packages/amap/index.html). Hierarchical clustering based on the distance matrix was calculated using the hclust function of the R package stats (R version 3.2.3).

#### Variance estimation

To estimate the variance explained by the host’s genetics and rumen bacteria, we used the REML approach in DMU [20]:

M1 $${\text{y}}_{\text{ijkl}} =\upmu + {\text{herd}}_{\text{i}} + {\text{parity}}_{\text{j}} + \left( {{\text{b}}_{1} \times {\text{DIM}}_{\text{k}} } \right) + \left( {{\text{b}}_{2} \times {\text{e}}^{{ - 0.05 \times {\text{DIM}}_{\text{k}} }} } \right) + {\text{g}}_{\text{i}} + {\text{e}}_{\text{ijkl}} ,$$

M2 $${\text{y}}_{\text{ijkl}} =\upmu + {\text{herd}}_{\text{i}} + {\text{parity}}_{\text{j}} + \left( {{\text{b}}_{1} \times {\text{DIM}}_{\text{k}} } \right) + \left( {{\text{b}}_{2} \times {\text{e}}^{{ - 0.05 \times {\text{DIM}}_{\text{k}} }} } \right) + {\text{m}}_{\text{i}} + {\text{e}}_{\text{ijkl}} ,$$

where ${\text{y}}_{\text{ijkl}}$ is the FA phenotype of individual ${\text{l}}$, $\upmu$ is the fixed mean effect, ${\text{herd}}_{\text{i}}$ is a fixed effect (${\text{i}} = 1, 2, 3$), ${\text{parity}}_{\text{j}}$ is a fixed effect (${\text{j}} = 1, 2$), ${\text{b}}_{1}$ and ${\text{b}}_{2}$ are the regression coefficient for ${\text{DIM}}_{\text{k}}$ ${\text{DIM}}_{\text{k}}$ is a covariate of days in milk (d3–d398) and is modelled according to Wilmink [21], ${\text{g}}_{\text{i}}$ is the random additive genetic effect of the animal, ${\text{m}}_{\text{i}}$ is the random effect associated with bacterial composition, and ${\text{e}}_{\text{ijkl}}$ is the random residual effect. The random effects are assumed to be independent normally distributed values described as follows: ${\mathbf{g}} \sim {\text{N}}\left( {{\mathbf{0}},{\mathbf{G}}\upsigma_{\text{g}}^{2} } \right)$, ${\mathbf{m}} \sim {\text{N}}\left( {{\mathbf{0}},{\mathbf{M}}\upsigma_{\text{m}}^{2} } \right)$ and ${\mathbf{e}} \sim {\text{N}}\left( {{\mathbf{0}},{\mathbf{I}}\upsigma_{\text{e}}^{2} } \right).$

The genomic relationship matrix (GRM) ${\mathbf{G}}$ was computed using all the SNPs based on the first method described by VanRaden [22]. The metagenomics relationship matrix (${\mathbf{M}}$) was computed based on the bacteria counts as previously described by Difford et al. [2]: ${\mathbf{M}} = {\mathbf{BB}}^{{\prime }} / {\text{c}}$, where ${\mathbf{B}}$ is the centered and scaled bacteria count matrix, and ${\text{c}}$ is the number of bacterial OTU.

The proportion of the total variance explained by the genetic effect of the animal (${\text{h}}_{\text{g}}^{2}$) was defined as:

$${\text{h}}_{\text{g}}^{2} =\upsigma_{\text{g}}^{2} /\left( {\upsigma_{\text{g}}^{2} +\upsigma_{\text{e}}^{2} } \right),$$

where $\upsigma_{\text{g}}^{2}$ is the genetic variation, and $\upsigma_{\text{e}}^{2}$ is the residual variation.

The proportion of the total variance explained by the effect associated with bacteria count (${\text{h}}_{B}^{2}$), which is also called microbiability [2], was defined as:

$${\text{h}}_{B}^{2} =\upsigma_{\text{m}}^{2} /\left( {\upsigma_{\text{m}}^{2} +\upsigma_{\text{e}}^{2} } \right),$$

where $\upsigma_{\text{m}}^{2}$ is the bacterial variation, and $\upsigma_{\text{e}}^{2}$ is the residual variation.

#### Cross-validation study to compare models

To assess the influence of the animal’s genome and of the metagenome on milk and FA traits, we performed a cross-validation study by using a two-step procedure. In the first step, phenotypic records on milk FA, PP and FP were adjusted for relevant factors (herd, parity, DIM) using the following linear model:

M3 $${\text{y}}_{\text{ijkl}} =\upmu + {\text{herd}}_{\text{i}} + {\text{parity}}_{\text{j}} + \left( {{\text{b}}_{1} \times {\text{DIM}}_{\text{k}} } \right) + \left( {{\text{b}}_{2} \times {\text{e}}^{{ - 0.05 \times {\text{DIM}}_{\text{k}} }} } \right) + {\text{e}}_{\text{ijkl}} ,$$

where ${\text{y}}_{\text{ijkl}}$ is the phenotype of individual ${\text{l}}$, and the factors are as for Model M1.

In the second step, we built a model in several steps for each adjusted phenotype (i.e. residuals obtained from Model M3) using the following linear mixed models:

M4 $$\tilde{y}_{i} =\upmu + {\text{g}}_{\text{i}} + {\text{e}}_{\text{i}} ,$$

M5 $$\tilde{y}_{i} =\upmu + {\text{m}}_{\text{i}} + {\text{e}}_{\text{i}} ,$$

M6 $$\tilde{y}_{ij} =\upmu + {\text{m}}_{\text{i}} + {\text{g}}_{\text{j}} + {\text{e}}_{\text{ij}}$$

M7 $$\tilde{y}_{ij} =\upmu + {\text{m}}_{\text{i}} + {\text{g}}_{\text{j}} + \left( {{\text{m}}_{\text{i}} \times {\text{g}}_{\text{j}} } \right) + {\text{e}}_{\text{ij}}$$

where $\tilde{y}_{ij}$ is the adjusted phenotype for each milk or FA trait, ${\text{g}}_{\text{j}}$ is the random additive genetic effect of the animal, ${\text{m}}_{\text{i}}$ is the random effect associated with bacteria count, ${\text{m}}_{\text{i}} \times {\text{g}}_{\text{j}}$ is the Hadamard product between ${\text{m}}_{\text{i}}$ and ${\text{g}}_{\text{i}}$, and ${\text{e}}_{\text{i}}$ (${\text{e}}_{\text{ij}}$) are the random residual effects. The random effects are re-estimated and are assumed to be independent normally distributed values described as follows: ${\mathbf{g}} \sim {\text{N}}\left( {{\mathbf{0}},{\mathbf{G}}\upsigma_{\text{g}}^{2} } \right)$, ${\mathbf{m}} \sim {\text{N}}\left( {{\mathbf{0}},{\mathbf{M}}\upsigma_{\text{m}}^{2} } \right)$ and ${\mathbf{e}} \sim {\text{N}}\left( {{\mathbf{0}},{\mathbf{I}}\upsigma_{\text{e}}^{2} } \right)$.

The analysis was performed using a genomic feature best linear unbiased prediction (GFBLUP) for each model separately, as implemented in the R library qgg (http://psoerensen.github.io/qgg/) [23, 24]. The features in Models M5, M6 and M7 are the bacteria count data and the genetic data (39,121 SNPs). The ability of each of the models (M4–M7) to predict phenotypes was assessed using a cross-validation procedure. The validation set was generated by randomly sampling 50 out of 292 animals and the remaining 242 animals were used as the training set. In the validation procedure, we estimated the model parameters based on the observations of the cows in the training data and predicted the phenotypes of the cows in the validation data. Then, we calculated the correlation between the predicted phenotype and the observed phenotype. This was repeated 10 times and the predictive ability of a model was defined as the average correlation of these 10 validations.

The predictive ability of each model was compared using a Welch’s t test (i.e. unequal variance t test) as implemented in R (R version 3.2.3). We tested whether the average correlation of these 10 validations was significantly higher than the average correlation obtained from another model. We performed the following comparisons: M4 versus M6 (bacteria are important); M5 versus M6 (genetics is important); M6 versus M7 (interaction between bacteria and genetics is important). A Bonferroni corrected P value lower than 0.05 was considered significant (n = 57 comparisons).

## Results

### Description of the milk data

The descriptive statistics for milk FA, FP and PP traits are in Table 1. Proportions in milk FA varied with the highest proportions being in C14:0 (11.60%), C18:1 *cis*-9 (20.27%) and C16:0 (31.24%), and the lowest in C13:0 (0.14%), CLA *cis*-9, *trans*-11 (0.48%) and C18:3 n-3 (0.53%). Coefficients of variation (CV) were highest for C14:1 *cis*-9, C16:1 *cis*-9, and C12:0, i.e. 33.97, 27.24, and 24.19%, respectively, and lowest for C16:0, C18:2n6, and C6:0 i.e. 10.92, 12.95, and 14.30%, respectively.

**Table 1:** Descriptive statistics of fat %, protein % and fatty acids in the milk of Holstein cows

Trait^a^	Mean	SD	CV (%)
Fat %	4.00	0.77	19.21
Protein %	3.33	0.35	10.37
Caproic acid (C6:0)	2.77	0.40	14.30
Caprylic acid (C8:0)	1.44	0.28	19.04
Capric acid (C10:0)	3.19	0.70	21.91
Lauric acid (C12:0)	3.69	0.89	24.19
Tridecylic acid (C13:0)	0.14	0.03	21.27
Myristic acid (C14:0)	11.60	1.95	16.81
Myristovaccenic acid (C14:1 *cis*-9)	1.00	0.34	33.97
Pentadecylic acid (C15:0)	1.11	0.24	21.42
Palmitic acid (C16:0)	31.24	3.41	10.92
Palmitoleic acid (C16:1 *cis*-9)	1.77	0.48	27.24
Margaric acid (C17:0)	0.57	0.11	19.11
Stearic acid (C18:0)	9.53	2.00	20.97
Elaidic acid (C18:1 *trans*-11)	1.35	0.30	22.29
Oleic acid (C18:1*cis*-9)	20.27	3.97	19.61
Linoleic acid (C18:2 n-6)	1.87	0.24	12.95
α-Linolenic acid (C18:3 n-3)	0.53	0.09	16.26
Conjugated linoleic acid (CLA *cis*-9, *trans*-11)	0.48	0.11	23.88

*SD* standard deviation, *CV* coefficient of variation

^a^Individual fatty acids given as the wt/wt%

### Rumen bacteria

The hierarchical cluster analysis on the 3055 rumen bacterial OTU (after filtering) did not show a clear pattern of OTU clusters. Among the OTU with high abundance, most of the OTU were assigned to *Bacteroidales* and *Prevotella* (see Additional file 1), whereas those with low abundance were mostly assigned to the two aforementioned groups and to *Clostridiales*, *Lachnospiraceae*, *Ruminococcaceae*, *Ruminococcus*, *Treponema*, and *RF39*.

### Influence of rumen bacteria and the host genetics on milk fatty acids

Table 2 shows the estimated heritability (${\text{h}}_{\text{g}}^{2}$) and microbiability (${\text{h}}_{B}^{2}$) for each trait based on M1 and M2, respectively. In general, ${\text{h}}_{\text{g}}^{2}$ was relatively high for all FA [ranging from 0.69 (C14:1 *cis*-9) to 0.11 (C18:1 *trans*-11; C18:1 *cis*-9)], except for C14:0 and C18:3 n-3, which had values close to 0. In general, ${\text{h}}_{B}^{2}$ was lower than ${\text{h}}_{\text{g}}^{2}$, and the main FA affected by rumen bacteria were C15:0, C17:0, C18:2 n-6, C18:3 n-3 and CLA with ${\text{h}}_{B}^{2}$ ranging from 0.26 to 0.42. For the other FA C6:0, C8:0, C10:0, C12:0, C16:0, C16:1 *cis*-9 and C18:1 *cis*-9, ${\text{h}}_{B}^{2}$ was close to 0. In all cases, the estimated standard errors were relatively large, which can be explained by the limited sample size (Table 2).

**Table 2:** Total variance explained by additive genetic^a^ (${\text{h}}_{{\rm g}}^{2}$) and rumen bacteria^a^ (${\text{h}}_{B}^{2}$) effects

Trait	${\text{h}}_{\text{g}}^{2}$	SE ${\text{h}}_{\text{g}}^{2}$	${\text{h}}_{B}^{2}$	SE ${\text{h}}_{B}^{2}$
Fat %	0.19	0.16	0.08	0.07
Protein %	0.18	0.14	0.08	0.09
C6:0	0.14	0.12	4.25e−07	0.05
C8:0	0.17	0.13	4.57e−07	0.06
C10:0	0.16	0.13	2.87e−06	0.08
C12:0	0.19	0.14	4.23e−07	0.07
C13:0	0.19	0.16	0.12	0.09
C14:0	2.45e−07	0.11	0.14	0.12
C14:1 *cis*-9	*0.69*	0.22	0.03	0.05
C15:0	*0.53*	0.21	*0.42*	0.18
C16:0	0.17	0.14	1.33e−07	0.07
C16:1 *cis*-9	*0.42*	0.19	3.63e−06	0.06
C17:0	0.22	0.14	0.30	0.16
C18:0	0.26	0.16	0.18	0.13
C18:1 *trans*-11	0.11	0.13	0.16	0.15
C18:1 *cis*-9	0.11	0.12	1.22e−08	0.06
C18:2 n-6	0.12	0.12	0.26	0.14
C18:3 n-3	2.17e−05	0.12	*0.31*	0.14
CLA *cis*-9, *trans*-11	0.24	0.14	0.33	0.17

Significant heritabilities and microbiabilities are in italic

*CLA* conjugated linoleic acid

^a^${\text{h}}_{\text{g}}^{2}$ and ${\text{h}}_{B}^{2}$ were estimated based on models M1 and M2, respectively

### Predictive ability of milk fatty acids

The predictive ability of each model is in Table 3. In general, predictive ability for individual FA was low regarding both the rumen bacteria component and the genetic component. The comparison M4 versus M6 shows that adding rumen bacteria information improved predictive ability significantly for C15:0 (P = 9.50e−07) and C18:3 n-3 (P = 8.81e−18), and only slightly for C13:0 (P = 0.07), C14:0 (P = 0.01) and C18:2 n-6 (P = 0.08). The comparison M5 versus M6 shows that genetic information had a more significant effect on improving predictive ability for C6:0, C8.0, C10:0, C12:0, C14:1 *cis*-9, C16:0, C16:1 *cis*-9 and C18:1 *cis*-9 (Table 2). Finally, the comparison M6 versus M7 suggests a slight improvement in predictive ability for C18:1 *cis*-9 (P = 0.003) when adding an interaction component between bacteria and genotype, but it was not significant after adjustment for multiple testing.

**Table 3:** Mean predictive ability^a^ (PA) and between brackets root mean square error of the prediction (RMSE) for models M4, M5, M6 and M7 and P value for the differences in PA between M4 versus M6, M5 versus M6, and M6 versus M7

Trait	Mean PA (RMSE) M4	Mean PA (RMSE) M5	Mean PA (RMSE) M6	Mean PA (RMSE) M7	P value M4 versus M6	P value M5 versus M6	P value M6 versus M7
Fat %	0.09 (0.75)	0.15 (0.75)	0.13 (0.75)	0.13 (0.75)	0.34	0.45	0.96
Protein %	0.14 (0.28)	0.03 (0.28)	0.11 (0.28)	0.12 (0.28)	0.39	0.02	0.84
C6:0	0.15 (0.37)	− 0.07 (0.37)	0.14 (0.37)	0.15 (0.37)	0.93	*3.70e*−10*	0.92
C8:0	0.15 (0.25)	− 0.12 (0.24)	0.15 (0.24)	0.15 (0.24)	0.97	8.15e−13*	0.97
C10:0	0.10 (0.53)	− 0.14 (0.53)	0.10 (0.53)	0.10 (0.53)	1.00	5.38e−11*	0.84
C12:0	0.17 (0.59)	− 0.10 (0.59)	0.16 (0.59)	0.16 (0.59)	0.89	2.70e−10*	0.94
C13:0	0.09 (0.03)	0.18 (0.03)	0.16 (0.03)	0.16 (0.03)	0.08	0.46	1.00
C14:0	− 0.03 (1.20)	0.08 (1.21)	0.06 (1.21)	0.04 (1.22)	0.01	0.54	0.61
C14:1 *cis*-9	0.37 (0.24)	0.06 (0.26)	0.37 (0.24)	0.37 (0.24)	1.00	4.94e−18*	1.00
C15:0	0.22 (0.14)	0.34 (0.14)	0.38 (0.13)	0.38 (0.13)	9.50e−07*	0.15	0.90
C16:0	0.12 (2.42)	− 0.17 (2.42)	0.11 (2.42)	0.10 (2.42)	0.92	1.81e−10*	0.80
C16:1 *cis*-9	0.23 (0.44)	− 0.11 (0.45)	0.23 (0.44)	0.23 (0.44)	1.00	3.10e−17*	1.00
C17:0	0.15 (0.08)	0.07 (0.08)	0.10 (0.08)	0.10 (0.08)	0.18	0.39	0.92
C18:0	0.16 (1.30)	0.07 (1.33)	0.12 (1.31)	0.12 (1.31)	0.23	0.19	0.96
C18:1 *trans*-11	0.07 (0.19)	0.02 (0.19)	0.03 (0.19)	0.03 (0.19)	0.24	0.87	0.93
C18:1 *cis*-9	0.11 (2.66)	− 0.13 (2.66)	0.11 (2.66)	0.00 (2.67)	0.99	4.15e−10*	3.20e−03
C18:2 n-6	0.09 (0.23)	0.16 (0.23)	0.15 (0.23)	0.15 (0.23)	0.08	0.71	1.00
C18:3 n-3	− 0.08 (0.08)	0.29 (0.07)	0.29 (0.08)	0.29 (0.08)	8.81e−18*	1.00	1.00
CLA *cis*-9, *trans*-11	0.15 (0.09)	0.16 (0.09)	0.14 (0.09)	0.13 (0.09)	0.77	0.56	0.81

*Significant after Bonferroni correction for multiple testing at the P < 0.05 level (n = 57 comparisons)

^a^Average correlation between predicted and real FA phenotype in the validation set over 10 replicates of the validation

## Discussion

In this study, we evaluated the influence of the bacterial composition of the rumen microbiome on the biosynthesis of specific FA in bovine milk. FA composition in milk is the result of a complex process in which FA originate from different sources. Short chain and medium chain FA are synthesized de novo in the mammary gland, while long chain FA derive mainly from feed [7]. In general, the heritability of de novo synthesized FA is high, whereas that of long chain FA is low to moderate [8]. Poulsen et al. [12] reported that the feeding regime has a clear influence on the composition of long chain FA, but also that they are modified considerably in the rumen and by desaturation in the mammary gland. Even if cows have the same feeding regime, individual feed intake per cow varies because its energy requirement differs depending on parity and lactation stage. Furthermore, cows sort out their feed even when fed a TMR. Thus, one can argue that the microbial composition in the rumen could be a proxy for feed type, rather than influencing milk fat composition. However, Sasson et al. [25] showed that abundance of some bacteria in the rumen is heritable [25]. Thus, variation in the rumen microbiome may influence the biosynthesis of milk FA and the modifications of feed-derived FA.

### Core microbiome

Henderson et al. [1] have suggested that a ‘core microbiome’ of dominant bacteria exists in the rumen at the genus level or higher and consists of *Prevotella*, *Butyrivibrio*, *Ruminococcus* as well as unclassified *Lachnospiraceae*, *Ruminococcaceae*, *Bacteroidales* and *Clostridiales*. Our results are in line with the results of Henderson et al. [1], i.e. they show that the clusters of OTU with the highest counts were mainly assigned to *Prevotella* and the unclassified group *Bacteroidales*, whereas the clusters with the lowest count data also contained the (unclassified) groups *Clostridiales*, *Lachnospiraceae*, *Ruminococcaceae*, *Ruminococcus*, *Treponema*, and *RF39*.

### Heritability, microbiability and sample size

In our study, estimated heritabilities for fat % and protein % were low (0.19, and 0.18, respectively) compared to previous reports on fat %, i.e. 0.24 in [9] and 0.47 in [10] and on protein %, i.e. 0.47 in [26] and 0.53 in [27]. The discrepancy between our current results and those in the literature could be due to the use of different models to estimate the variances or simply to the relative small sample size used here. Ideally, the variance parameters in this study would be estimated jointly, combining models M1 and M2, but the sample size in our study limits the possibility of estimating parameters from the full model in which both ${\mathbf{G}}$ and ${\mathbf{M}}$ are fitted simultaneously. Nevertheless, we believe that our results show that some of the milk FA are affected by the rumen microbiome, and that our study could serve as a basis for future studies on the influence of the microbiome on milk FA composition.

Our results show that the short and medium chain FA have a stronger genetic component, compared to the long chain C18 FA, which is in line with the expectation that long chain FA derive from feed. However, our results do not confirm the trend that saturated FA have a higher heritability than unsaturated FA as previously reported [9, 10]. Comparison of our results to an earlier independent study on milk FA composition in Holstein cattle [9] indicates that the estimated heritabilities are in line with those in [9] except for C14:0 and C18:3 n-3, for which we detected no genetic component. Interestingly, both C14:1 *cis*-9 and C16:1 *cis*-9 had high heritabilities, which reflect a strong genetic influence related to the desaturase activity in the mammary gland. The conversion of C14:0 and C16:0 into their mono-unsaturated counterparts is catalyzed by stearoyl-CoA desaturase 1 (SCD1). The SCD1 gene is located on *B. taurus* chromosome (BTA) 26 and is associated with milk FA composition [28].

We detected no influence of the rumen bacteria composition on the C6–C12 and C16 FA group, which suggests that they have little or no effect on de novo synthesis in particular, but also on C16:0 derived from feed. This is further reflected by the absence of influence on C14:1 *cis*-9, and C16:1 *cis*-9. In general, the influence of bacteria in the rumen on the formation of milk FA is more pronounced for the odd-chain FA (C15:0, C17:0) and polyunsaturated C18 FA (C18:2 n-6, C18:3 n-3 and CLA *cis*-9, *trans*-11), as expected based on the origin of the FA in milk [29]. These FA originate partly from hydrogenation processes in the rumen by specific microorganisms that affect feed-derived C18 FA and partly from odd-chain FA that are mainly synthesized microbially in the rumen [30].

For C17:0 and many of the C18 FA, the variation in rumen bacterial content explained more of the host phenotypic variation than the host genome variation, albeit marginally, and with a large standard error. Although variation in these FA is often associated with differences in the feed, in our study, feed variation was minimized since animals were fed a standardized TMR. Furthermore, these findings confirm the existence of variation in the host genome and that the microbiome can explain a part of the variation in some complex host traits. This agrees with the findings of a study that investigated the influence of gut bacteria in 207 pigs and estimated the proportion of variation explained by ${\text{h}}_{B}^{2}$ and ${\text{h}}_{g}^{2}$ for feed intake (${\text{h}}_{B}^{2}$ = 0.16, ${\text{h}}_{g}^{2}$ = 0.42), daily gain (${\text{h}}_{B}^{2}$ = 0.28, ${\text{h}}_{g}^{2}$ = 0.11) and feed conversion ratio (${\text{h}}_{B}^{2}$ = 0.21 and ${\text{h}}_{g}^{2}$ = 0.19) [31]. That study also showed that the abundance of 49 genera of the pig gut microbiome was heritable [31].

### Prediction of fatty acids in the milk

Recent findings in dairy cattle suggest that the host genetics has some influence on the rumen microbiome [25, 32, 33]. There is considerable interest in understanding the interactions between the host and the rumen microbiome for directing desired changes to the host phenotype. If associations between heritable rumen bacteria and phenotypes of the host exist, there is scope for research in selective breeding. Selective breeding takes generations to induce genetic changes in livestock populations, whereas interventions on the rumen microbiome can act rapidly within a few generations. Rumen transfaunation using the cud from a healthy donor cow to treat a recipient cow with a digestive disorder has long been an effective method of implementing a desired change to the host cow phenotype [34]. Recent work with repeated inoculations in beef heifers on a poor straw-based diet with rumen contents from bison has shown that the rumen bacteria and the host metabolic activity are altered, which result in increased protein and nitrogen retention [35].

It is relevant to investigate whether the bacterial and genetic information can contribute to predicting the milk fat composition of cows. In general, we found that the predictive value for FA was low, which could be due to the relatively small sample size used in our study. Genotype information is of higher value for the prediction of specific FA in the milk than information on the rumen bacterial composition. We found that genotype information was relevant for the prediction of the medium chain FA C6 to C12, C14:1 *cis*-9 and C16:0, C16:1 *cis*-9. This is in line with the fact that these FA are fully or partly de novo synthesized or reflect desaturase activity in the mammary gland.

Interestingly, information on the rumen bacterial community was better at predicting the C15:0 and C18:3 n-3 content in milk (r = 0.29–0.34) than genotype information. The predictive value of rumen bacteria for C15:0 and C18:3 n-3 was in line with that of gut bacteria in pigs for feed intake, daily gain and feed conversion (r = 0.33–0.41) [30] and that of methane emission in dairy cows using full rumen metagenomics sequences (r = 0.466) [35]. The estimated ${\text{h}}_{g}^{2}$ and ${\text{h}}_{B}^{2}$ for C15:0 were similar, which is as expected since C15:0 is known to be both synthesized de novo and derived from blood [36, 37]. However, information on the microbiome is preferable for predicting the C15:0 content in milk. Regarding the prediction of C18:3 n-3 content in milk, C18:3 n-3 derives from the feed and thus depends highly on the feeding regime of the cow [37] and on the microbiome, which plays an important role in degrading the feed in the rumen and thereby regulates the C18:3 n-3 content in milk through rumen biohydrogenation. Our results also show that, when ${\text{h}}_{g}^{2}$ or ${\text{h}}_{B}^{2}$ are equal to 0 (Table 2), the mean predictive value is negative (Table 3), which indicates that the correlation between the observed and predicted phenotypes are biased downwards.

## Conclusions

Our findings show that variation in rumen microbiome composition has a pronounced influence on the content of odd chain FA and the polyunsaturated C18 FA, and to a lesser extent, on the content of the short and medium chain FA in milk. The prediction of individual FA content in milk based on information of either the animals’ genotypes or the rumen bacteria was low. The results can be explained from a biological point of view, e.g. the ${\text{h}}_{g}^{2}$ for the saturated FA was generally higher than ${\text{h}}_{B}^{2}$ and the predictive ability of the model fitting the GRM was better for the saturated FA than the model fitting the microbial relationship matrix. Nevertheless, standard errors of the heritability estimates were large and, in some cases, the predictive values tended to be biased downwards, which indicated that it is necessary to increase the sample size in a future study.

## Additional file

**Additional file 1.** Annotation of the most abundant bacteria OTU.
